# Selections and Abstracts

**Published:** 1904-01

**Authors:** 


### SELECTIONS AND ABSTRACTS.

---

"EDUCATION AGAINST PULMONARY TUBERCULOSIS."*

In presenting this short paper to you it would be presumptuous to suppose that I could offer any new ideas or impart any knowledge to this body of medical men upon a subject that has been studied so thoroughly, and my only object in asking your attention is to bring forcibly to your minds some of the facts relative to that most dangerous communicable disease—pulmonary tuberculosis.

The relation of human and bovine tuberculosis; questions of heredity; the importance and advisability of institutional treatment under State supervision; methods of transmission other than from sputum of the patient, and all other aspects of the tuberculosis question will be avoided and only the results of education of the public be discussed.

Within the last year or two the whole world has been waking up to the truth. The great congress of experts at Berlin and later at London are but signs of the cosmopolitan interest in the disease.

The four following facts present an epitome of the conclusions arrived at by these experts:

1. Tuberculosis is a communicable disease, due to Koch's tubercle bacillus acting on an organism prepared to receive it, or unable to resist the bacilli when present in large numbers.

2. Tuberculosis is not to any great extent hereditary.

3. Tuberculosis may be prevented by reducing the sources of infection, by improving the environment, by strengthening the individual.

4. Tuberculosis, in many even of its severest varieties, can be cured.

These propositions may now be accepted as scientific truths and will form the working plan of all future efforts directed against

---

*Read by Claude C. Pierce, M.D., Assistant Surgeon U. S. P. H. and M. H. S., etc., Mullet Key, Fla., before the Florida State Medical Association, April, 1903.

tuberculosis. When Koch read his paper before the British Congress of Tuberculosis in July, 1901, he said:

"Strictly speaking, the fact that tuberculosis is a preventable disease ought to have become clear as soon as the tubercle bacillus was discovered and the properties of this parasite and the manner of its transmission became known. But the strength of a small number of medical men was inadequate to the conflict with a disease so deeply rooted in our habits and customs. Such a conflict requires the co-operation of many, if possible of all, medical men, shoulder to shoulder with the State and the whole population, and now the moment when such co-operation is possible seems to have come."

That the medical profession is thoroughly aroused to their opportunities and responsibilities in preventing this death-dealing disease is evidenced by the many articles that have recently appeared from all parts of the world and by the great work which has been done in securing legislation for providing proper sanatoria for the treatment of those afflicted with consumption, for the education of the people as to the means of transmission, for maintaining surveillance of patients in crowded sections, for the destruction of sputum, and such measures as limit the means of spreading the great white plague.

That the profession is receiving the co-operation of the public, witness the organized efforts of national, state and municipal governments and independent societies in their work of prevention and education. The general government has enforced a measure to diminish the foci of infection by excluding tuberculous aliens from our shores.

While this has been criticised as harsh by some, it has the support of many of the most thoughtful sanitarians, and the act itself has done incalculable good by its effect in arousing discussion and spreading knowledge of the method of transmission.

There is now a society in New York City which gives free lectures to the public upon the nature, cause and prevention of consumption, and distributes literature to those afflicted. This is in addition to the admirable system of reporting and tabulating of all cases under the auspices of the city board of health, the proper instruction of the patient upon the necessity of destruction of sputum and the disinfection of quarters occupied, upon the death or removal of the sufferer.

In Ohio, there is a Tuberculosis Commission, under the auspices of the State, which does work similar to the New York City Board.

In Michigan, the work has been carried into effect most thoroughly under the direction of Dr. Baker, the State Health Officer, by means of monthly "Teachers' Sanitary Bulletins," which disseminate knowledge, not only of tuberculosis, but of all communicable diseases.

Similar work is being done in Rhode Island, Maine, Indiana and other States by the Boards of Health.

When we remember that practically thirteen per cent. of all deaths in the United States is caused by tuberculosis and that nearly every case could have been prevented by proper measures, the enormity of our loss in money and valuable lives from this avoidable cause will surely enlist the earnest efforts of all humanitarians in a relentless war against the vegetable parasite which robs so many homes of bread-winners and the State and county of loyal citizens.

The great importance which was formerly attached to the hereditary transmission of the disease tended to prevent active work in prophylaxis, and all the energy of medical minds was devoted to therapeutic measures.

Now that we know that practically none actually inherit tuberculosis, and that the usual way of contracting the disease is by the direct introduction of germs from the sputum of patients, we have a definite working basis, and the question of eradicating tuberculosis from our country resolves itself into the destruction of bacilli by the proper treatment of the expectoration of those already infected with the disease.

What is being done elsewhere to limit the ravages of tuberculosis is our duty to see carried out here, as the entire country should educate the laity upon matters of sanitation. The very fact that the climate of our State is vastly more favorable to those suffering with pulmonary diseases than is that of most of the Northern States makes it all the more important that this work be taken up, as it is generally known that many suffering with tuberculosis seek health in the mild and genial air of Florida, and whether they are benefited or not depends upon the stage of the disease and condition of the patient at the time of his arrival as well as upon the location and subsequent treatment.

Furthermore, it is these incipient cases, where the patient is able to follow his usual avocation, or at least to walk about the streets, hotels and other public buildings, that disseminate most widely by promiscuous expectoration the germs capable of infecting others with consumption.

The great importance of preventing this unregulated spitting is impressive when we remember that it has been estimated that an ambulant phthisic will throw off seven billion germs in twenty-four hours. I can do no better than to quote Dr. Baker, State Health Officer of Michigan, to illustrate the necessity for and great benefit resulting from proper education. Dr. Baker says:

"Of all communicable diseases, consumption (pulmonary tuberculosis) is now the most dangerous. More people contract that disease than any other. Therefore, anything, any statement or any influence which belittles the importance of restricting the spread of consumption, does damage in the most vital point to the interests of the public health and safety.

"Improper housing and improper feeding of the poor are important evils to be done away with, because they lead to discomfort and lowered vitality and tend to spread disease. But if the germs of tuberculosis were generally restricted, any amount of lowered vitality because of improper housing and improper food would not cause a single case of consumption.

"The essentials for the restriction of consumption are: First, the general recognition of the truth that consumption is the most dangerous communicable disease. Knowledge of that fact is the power without which consumption can not be restricted. It is lack of action because of ignorance of this great truth—that consumption is spread from infected persons—that kills off the improperly housed and improperly fed poor. It is ignorance of this great truth that kills off the rich by tubercular disease, in spite of proper housing and proper feeding.

"It is the slow but gradual gaining of that precious knowledge by the common people, and action governed by that knowledge, that is reducing the mortality from consumption as it is being reduced in Michigan and other States.

"In order to be most useful to the public, it is essential that this important knowledge shall be gained by and shall govern the action of every coughing consumptive, who otherwise is a constant source of danger. Therefore, the consumptive should be promptly put in possession of that knowledge. This first essential can not be fulfilled by the public unless every case of well-developed consumption shall be reported to the health officer. Every case reported should be promptly informed how to avoid reinfection of the patient and spreading the disease."

Carefully compiled statistics show that the death rate of tuberculosis has declined in Michigan from 117 per 100,000 in 1881 to 83 in 1900.

"If the decrease continues at the same rate, the disease will be so restricted as to be practically extinct in Michigan in the year 1950. It is hoped that the decrease shall soon be at even a faster rate, and there is reason for that hope in the fact that the decrease has apparently resulted from the education of the people generally to the knowledge that consumption is a dangerous communicable disease, which may easily be restricted. It is, apparently, one more forcible illustration of the fact that 'knowledge is power.' In this instance, knowledge of the modes whereby consumption is usually spread, and of the ease with which its spread may be lessened, by the destruction or disinfection of all consumptive sputa, has apparently supplied a 'power' which has caused an unprecedented reduction in the death-rate from consumption. The extent of the 'campaign of education' which, in Michigan, began in 1880, and which took on an especially vigorous activity in 1891, can hardly be realized without a study of its history."

In conclusion, I desire to suggest the following measures for advancing the "Campaign of Education," which should be earnestly recommended by all medical men as matters for suitable legislation before city councils and the State legislature:

1. The enactment of the following ordinances by the city councils throughout the State, to discourage indiscriminate expectoration of consumptives, and all others as well: "No person shall spit on any sidewalk, nor in any church, hotel corridor, theater, grocery or market." "No person shall spit in any railway station, public building, steamboat, railway car, street railway car, or licensed vehicle, unless in spittoon or other receptacle." "Any person who shall violate any provision of sections——to ——, inclusive, of this chapter or section, shall be fined not less than $5.00 for the first offense, nor less than $20.00 for each and every subsequent offense."

Boston, Buffalo, Denver and other cities have similar anti-spitting laws.

2. The enactment of a law or insertion of an amendment to the law existing requiring the reporting of contagious diseases, making it the duty of the attending physician or head of the household to report cases of tuberculosis of the respiratory organs. An ordinance of the following form is suggested: "It shall be the duty of every physician called to attend a person sick or suspected to be sick with cholera, yellow fever, smallpox, chicken-pox, diphtheria, scarlet fever, measles, whooping cough, typhoid fever, typhus fever and tuberculosis of the respiratory organs, within twelve hours thereafter, to report the name and residence of such person to the Board of Health, or its proper officer, within whose jurisdiction such person is found; and where a person is taken sick with any of the above-named diseases, and a physician is not called, it shall in like manner be the duty of the owner or agent of the building in which such person resides, lives or is staying, and of the head of the family in which such disease occurs, to report the name and residence of the patient to the Board of Health or its proper officer."

Cincinnati has the above law.

3. The enactment of the following measures in proper legal phraseology as suggested by Dr. Formento, of Louisiana, carrying with the enactment a small appropriation, necessary for the increased expense caused by its provision:

(A) "It shall be the duty of all Boards of Health, as soon as notified of the existence of a case of tuberculosis, to take all proper precautionary measures, whether applied to the individual or to the house itself, against the spread of the disease.

(B) "Health Boards should prepare and distribute to all interested parties circulars containing instructions in regard to the sanitary management of the patient, the sick-room, the family and surroundings. The co-operation of the attending physician is to be greatly desired, and should be solicited in every case. Education and persuasion will accomplish more than coercive measures.

(C) "A proper registration of all cases, with the sanitary history of the infected house, as far as practicable, should be kept by the sanitary authorities, but in no case be made public.

(D) "Upon the death or removal of a patient, the quarters vacated shall be thoroughly disinfected in an improved manner, by an employee of the Board of Health."

4. The enactment by the School Boards of the State, counties and cities of the following law: "There shall be taught in every year in every public school in Florida the principal modes by which each of the dangerous communicable diseases is spread, and the best methods for the restriction and prevention of each such disease."

Such a law was passed in Michigan in 1895.

All the above measures are now in force in various localities and are limiting the ravages of tuberculosis, and I trust that the profession in our State will not be backward in inaugurating and enforcing measures which can accomplish so much for the prevention of communicable diseases.—*Mobile Medical and Surgical Journal.*

---

### HYDROPHOBIA, WITH REPORT OF A CASE.

BY JUDSON A. HULSE, B.S., M.D., AKRON, O., Anesthetist to City Hospital.

The word hydrophobia is from the two Greek words, hudor (water) and phobos (dread), meaning dread of water. Synonym, rabies.

An acute infectious disease of animals dependent upon a specific virus and communicated by inoculation to man.

*History.*—The disease was known to the ancients, including the

East Indians, Egyptians and Israelites, but is not mentioned by Hippocrates, though Democritus, living about the same time, is said to have considered it an affection of the nerves, allied to tetanus. Aristotle, however, recognized it and the Latin poets, Virgil, Homer and Ovid mentioned it, as did also the historian Plutarch.

*Etiology.*—It is a very rare disease. Indeed there are intelligent men in our profession who doubt its existence. Dr. Wilkes, of Guy's Hospital, London, has seen but three cases in more than thirty-five years' service, and our own Osler has seen but two cases in all his extended experience.

Rabies is capable of spontaneous development in the wolf, cat, dog, fox and skunk. In the Western States the skunk is especially liable to the disease. The Pasteur Institute at Chicago has treated fifteen persons who were bitten by skunks.

The period of incubation varies, the average being about two months, though claimed after three months have elapsed patient may be considered safe. It is shorter in children than in adults and in wounds about the face and extremities. Punctured wounds are the most dangerous. Bites from wolves are the most liable to communicate the disease to man, about 40 per cent. of bites from this source resulting in rabies, while only 15 per cent. develop the disease from the bites of rabid dogs.

The pathology is confined to lesions in the nervous system. Briefly, the morbid anatomy consists of an acute inflammation of the cord with destruction of the nerve elements, resulting in a hyperplasia of the neuroglia to replace them. Often there are no discoverable changes.

*Symptoms.*—The patient is depressed and melancholy. There may be irritation and numbness in the region of the bite. Wakefulness and a sense of impending danger are prominent and early symptoms. Increased sensibility and some slight difficulty in swallowing liquids are now manifest. All the symptoms become aggravated as the case advances, until violent reflex spasms develop, affecting the muscles of the larynx, pharynx and mouth, rendering deglutition difficult and at times impossible. An extreme degree of excitability now manifests itself and the mere sight of water, draughts of cool air, or sudden noises produce spasms. The mind is clear in the intervals, but maniacal symptoms are present in some cases. The pulse is rapid and the temperature ranges from 101 to 103. The spasms are accompanied by intense dyspnea and extreme mental pain, as evidenced by the terrible expression of agony and despair in the unfortunate victim's countenance. After from one to three days of suffering the paralytic stage develops, the spasms subside and death occurs from heart paralysis.

*Diagnosis.*—The only diseases with which hydrophobia is likely to be confounded are tetanus and hysteria. But the history of the bite and period of incubation, together with the set of muscles affected, will quickly settle the diagnosis.

From hysteria it is more difficult, but the absence of febrile conditions and a knowledge of the fact that the animal which inflicted the wound was not rabid will clear the diagnosis.

*Prognosis.*—Fatal in every case.

*Treatment.*—Among fanciful methods of treatment there is the madstone. If it adheres virus is present, if it becomes coated green the virus is extracted, and so on. Raw livers of dogs have been fed to patients and brains of rabid rabbits have been suggested as therapeutic food. In India entrails of an insect are administered, which cause bloody stools.

Immediately upon being bitten by a supposed rabid animal a ligature should be applied upon the limb above the wound and the wound cauterized by burning with nitric acid. The wound should not be closed, but kept freely open and allowed to bleed as much as it will. The animal should be kept under lock and key for at least one week and closely observed for signs of madness. If at the end of that time it is well there is no danger for the person bitten. When the disease is once manifest the patient should be kept in a dark room free from noise or annoyance of any kind. Restraint is not usually necessary. Nutrient enemata should be employed and water in large quantities given by the rectum. During the convulsions chloroform should be administered by inhalation. Chloral and the bromides are useful, as is also morphine hypodermically.

*Preventive Inoculation.*—The work of Pasteur in developing the treatment of rabies by preventive inoculation constitutes by far the most important addition to our knowledge of the nature of the affection and the possibilities of its cure. The therapeutic material used is a portion of the spinal cord of a rabbit that has died of hydrophobia. This is rubbed up in sterile cool water. These spinal cords have been dried over caustic potash, which diminishes the amount of virus but does not attenuate it. The virus is in a condition termed fixed. This is a virus which, inoculated subdurally in a rabbit, causes rabies in six days. In order to attain this condition of fixation it is necessary to inoculate from rabbit to rabbit until it has passed through about fifty. In the first inoculation rabies does not develop until the completion of 11, 12 or 15 days. The medulla oblongata is the part used in the inoculation of animals. Pasteur demonstrated its harmless efficacy. The method of treatment is subcutaneous hypodermic injections. The abdominal region is the part selected. The inoculation is begun with the cord that has been preserved for fourteen days; the second day, cord thirteen days old is used; the third day, the twelfth-day cord, etc., until the fifth-day cord is reached, when a new series of inoculations is commenced with the cord of the ninth or tenth day. In the intensive method the inoculations of the morning of the first day are from cords of the fourteenth and thirteenth days, rubbed up together, cords of twelfth and eleventh days being used the same evening. Cords of tenth and ninth days the following morning and cords of the eighth and seventh days the same afternoon. On the third day, cord six days old is used; on the fourth, cord five days old; on the fifth, cord four days old; on the sixth day cord three days old is used. Then a new series is begun with cord five days old.

After from one to seven weeks' treatment the patient is regarded as immune and the subsequent development of rabies is but slightly to be feared, as the mortality is but one-third on one per cent. in patients so treated.

I am indebted to Dr. N. G. Kierle, director of the Institute at Baltimore, and to Dr. A. Lagonire, of the Chicago Pasteur Institute, for much valuable information, which they kindly furnished me for use in the preparation of this paper.

REPORT OF CASE—HYDROPHOBIA.

Miss Nora Tuttle, age 14, of Courtland, Ohio, my old home, a girl whom I had known from her childhood, was visiting at my home last summer. Two months previous to her visit she was bitten through the left ear by a strange pup which, to all appearances, was playing with her baby sister on the floor. She was bitten while in the act of stooping to take the child away. No physician was called, but the wound was disinfected with dioxygen, and it healed quickly and kindly. The family joked about the affair at the time and the incident was forgotten. She came to our home about the first of July, and seemed well and happy, although at times she had momentary sensations of evil forebodings, which, while noticed by myself and other members of the family, elicited no anxiety on our part because of the girl's rapid growth and nervous temperament. On Tuesday, July 15, she complained of a stinging sensation in the bitten ear, followed by numbness. I examined it and found some congestion and blueness. Headache and pain in left side of neck were now present, which the usual remedies failed to relieve, but which was finally relieved by the use of moist cloths, which when applied caused nervous startings. She was wakeful Tuesday night, but seemed all right Wednesday, though for some reason which she could not explain did not drink any liquids. Though thirsty, she would go in where the water was kept but return without touching it. She spoke of the wound and the dog and the fear of death from hydrophobia. I calmed her fears in this regard and she never referred to it again. Thursday she seemed improved, but drank no water. Accompanied by my mother she went shopping and appeared much improved mentally, laughing and joking, but did not sleep Thursday night. Friday she seemed well enough, and tried to drink water but could not. During supper, in an attempt to drink tea, she was seized with the first convulsion, which was violent but of short duration. It frightened her, and as soon as she recovered her breath she ran screaming to my mother. She became very nervous now, but had no more convulsions until 9 p. m., when they came on in rapid succession. The convulsions were so violent that it required two persons to restrain her. She was spitting constantly. By 10 o'clock the nervecenters had become so irritable that the mere mention of water was sufficient to cause a convulsion. The temperature was 102° and the mental agony extreme. During the attack she would seize us and hold on like a vise, becoming maniacal, but as soon as the spasms had subsided would express a pitiful concern, fearing she had hurt us. Dr. C. W. Milliken now saw the case with me, and kindly took charge of it. He ordered 120 gr. bromidia per rectum and ice to allay thirst. She managed the ice quite well, and declared it was a great comfort to her. To the most imaginative mind Dante's vivid description of hell could not picture anything that would equal the intense mortal fear and anguish now expressed in the girl's eyes as she lay trembling like a leaf in anticipation of death in a form she knew not. After the administration of the bromidia she became quiet, the convulsions ceased and she fell asleep and slept well the entire night. The following morning the convulsions were resumed, being preceded by hallucinations. Dr. Milliken had not yet come in, and I administered more bromidia per rectum and chloroform by inhalation until the convulsions ceased. She was now spitting frothy mucus constantly—the so-called "cotton spitting"—and it was profuse. About 9 a. m. her general condition had very much improved, the convulsions had entirely ceased. She sat up and partook of a light breakfast, and was rational and even cheerful in her demeanor. The pulse remained weak and rapid, and she died suddenly of syncope at about 10 a. m.

The principal points of interest in this case it seems to me were:

1. The evil forebodings for weeks in advance of any other symptoms.

2. The peculiar stinging with congestion in the wound.

3. The intense hyperidrosis, clothes wet.

4. The fact that ice can be used to allay thirst when water can not.

5. The marvelous power of bromidia in controlling the convulsions and inducing sleep.

A very rare disease, the text-books say, and so it is, but to him who has been called upon to stand helplessly by and witness the death throes of a friend begging piteously for relief, which he can not give, the study of the disease assumes a new aspect, and the work of that great scientist, Pasteur, stands out to him not so much in the light of science, but as the work of an unselfish life given in the noblest service to suffering humanity.—*Columbus Med. Journal.*

---

### DIET AND DRUGS IN NEPHRITIS.*

Although the kidney is comparatively small in size, its blood supply is large and its function important. It has an excreting and probably an internal secreting work to perform. It is a long-suffering organ and has a heavy burden to bear. Its health depends, in a great measure, upon the normal metabolic and depurative work of the liver, upon the state of the genito-urinary tract below the upper opening of the ureter, upon the cutaneous circulation and vasomotor influences, and upon the absence of pressure and traumatism. Disease of the kidney may rapidly lead to destruction of life, or may persist for years without producing symptoms sufficiently marked to attract the patient's attention. On the other hand, the early restoration of function in some of the gravest cases of acute nephritis, and the slow but steady and resistless progress towards a fatal termination of most of the chronic cases, impress upon the physician the importance of prompt diagnosis and treatment.

Inasmuch as the fundamental principle in the treatment of all diseased organs is to secure rest, and inasmuch as the work of the kidney is to eliminate certain ingredients of the blood the retention of which would be injurious, it is essential in the management of nephritis, not only to maintain a normal composition of the blood, but at times to so change the quality of the blood as to reduce its excretion products to a minimum. Syphilis, lead-poisoning, and other infections or toxic conditions require in addition a specific or special treatment.

Since the quality and quantity of food and the use of alcoholic stimulants are acknowledged to be frequent and potent factors in the production of renal diseases, the matter of diet in nephritis is

---

*Read by William S. Gordon, M.D., Richmond, Va., Professor of Principles and Practice of Medicinê, University College of Medicine, Richmond, Va.; Visiting Physician to Virginia Hospital, etc., before the Medical Society of Virginia, at Roanoke, September 15, 1903.

to be esteemed of prime importance. Dieting is not what the patient so often supposes it to be—starvation—but a regulation of food in such a manner as to rest the kidneys and at the same time adequately nourish the body. Nor should it be forgotten that a diseased kidney must be fed, and that no injury must be inflicted upon it by withholding what is required for its own vitality and the performance of any function at all. This truth necessitates a distinction in the treatment of acute and of chronic nephritis; in the former of which diseases complete abstinence from food for a while may be of great service, whereas in the other condition the blood must be enriched and the kidney nourished while its work is diminished.

Apart from the views of other observers, my own experience leads me to express the belief that gout and lithemia, in whatever manner produced, are responsible for a large number of cases of nephritis. Those of us who have studied our cases carefully and watched the effect of irritants in the blood or urine can not escape the conviction that excessively acid urine, excess of solids and especially the presence of crystals of uric acid and calcium oxalate can not fail, in the long run, to injure the delicate epithelium of the tubules and to stimulate the growth of connective tissue. In treating nephritis, therefore, it follows that a diet which promotes a normal reaction of the urine and prevents the formation of abnormal ingredients is clearly indicated.

Having attempted to lay down general dietetic principles, let us bestow some attention upon special articles of food. I recall a case of which a brother practitioner spoke to me, that of a young child suffering with acute nephritis, drinking a large amount of milk as an exclusive diet, but still losing a decided amount of albumen. I advised a diet of water and carbohydrates for several days, thus reducing to a minimum the amount of urea excreted. The result was prompt improvement. I have recently had under my care a little girl who had scarlet fever a year ago, and who subsequently developed nephritis. Intermittent albuminuria persisted after the casts had disappeared. The milk diet adopted by the first physician who treated the case may have been serviceable in the acute stages, but the child was left anemic and the albuminuria was not relieved. I discontinued milk, allowed, with vegetables and fruits, chicken and eggs in moderation, administered iron, and sent the child to the mountains. At last accounts the albumin had disappeared and the patient was steadily improving. A third case is instructive—that of an elderly woman with a long-standing case of nephritis. I advised a diet mostly of milk. Constipation resulted and the albumin and casts increased. Complete flushing of the rectum and lower bowel caused a marked diminution of the albumin and, practically, a disappearance of the casts. These cases convey their own lessons and teach that an exclusive milk diet, so often recommended for nephritis, may do harm by being abused and furnishing too much urea, by causing constipation and absorption of toxins, by failing to supply the blood with sufficient nourishment, or by setting up lactic or butyric fermentation. The diet should be suited to the case, and a routine diet for all cases is unscientific and should be avoided.

On the other hand, we are all familiar with the benefit derived in a large number of cases of nephritis from a diet composed wholly or largely of milk. Abstinence from meats, alcohol, tea, coffee and cocoa, and a partial or complete reduction of sweets and acids, with the use of alkaline waters, rarely fail to produce prompt and beneficial results, especially in acute cases and in the first stages of the chronic cases. Haig claims that gout is not primarily due to excess of acids and sweets, but that these article, do harm by causing acid fermentation engrafted upon pre-existing conditions due to excess of proteid food. However, this may bes I am convinced that a large number of cases of nephritis, lithemia, gout, oxaluria, and other allied abnormal states are not cured or held in abeyance merely by cutting off the main articles of food or drink which Haig enumerates as the causes of uricacidemia. It is true that nephritis, once established, changes the composition of the blood and consequently of the urine; but it is all the more true that abnormal blood leads to abnormal urine, and that abnormal urine leads to renal diseases. My own rule is so to regulate the patient's diet as to maintain a normal urine; and so long as the urine is normal the kidney will, in the large majority of cases, be healthy or show marked improvement. Even secondary causes will, under these circumstances, be for the most part inoperative. So much for general principles. The careful practitioner will make special adaptations and note his results by repeated urinary examinations.

There is one point regarding nitrogenous food which ought to be emphasized, and this is that fowl, game, fish and eggs should not be indulged in to excess, because the patient has been prohibited the use of butcher's meat. There are cases in which all of the above-mentioned articles have to be cut out of the dietary. The injurious effects of eggs have been recently impressed upon me by the history of one of my cases. The young man was markedly lithemic and for years had been the subject of an obstinate psoriasis. After months of treatment, largely dietetic, he returned to me with samples of urine which showed a specific gravity of 1036, and which contained an abundant precipitate of calcium oxalate. The eggs, which he acknowledged having freely eaten, were prohibited, and in ten days the urine was in every respect normal.

Although I believe that hygienic measures are of paramount importance in the treatment of nephritis, I am not a skeptic on the subject of drugs. The more we study drugs and become familiar with their physiological action, the more should we be convinced of their therapeutic value when intelligently employed. Their action is governed by certain laws, which, in a measure, we are supposed to understand; and leaving out idiosyncrasies and unreliable preparations of medicinal agents, we may reasonably expect more or less success from an application of our therapeutics based upon the pathology and the correct diagnosis of the case in hand.

The value of diuretics in acute nephritis, when urinary secretion has almost or entirely ceased, is a contested point. Theoretically, they may be contraindicated in many cases, and high authorities advise against them; but theories and the dictum of authorities often wane before facts. I can vividly recall two cases of scarlet fever occurring years ago in my father's family, followed by severe nephritis and anasarca. The good old family doctor made an acetic extract of iron—so to speak—by dropping rusty nails into vinegar and drenching his patients with the potion. And I shall never forget how busy the attendants were kept carrying off the dropsy, and how rapidly the sick ones recovered. A year or two ago I was called in consultation to a man suffering from acute parenchymatous nephritis. The kidneys had apparently stopped working, and nothing but the profuse diaphoresis maintained by the attending physician saved the patient from fatal uremia. We concluded to give infusion of juniper and bitartrate of potassium. The result was magical; free diuresis set in, and the case rapidly went on to convalescence. Such happy results are often obtainable in chronic cases when the tubules become blocked. Irritating diuretics may do harm, but we are fortunate in possessing many agents which need not be mentioned but which are non-irritating and productive of the greatest benefit. In this connection, it is well to remember that an efficient cholagogue administered before the diuretic or in conjunction with it is a powerful auxiliary in the treatment. Free biliary secretion and open bowels are frequently sufficient to produce a prompt change for the better in the patient's condition.

The usefulness of diaphoretics, particularly in acute cases of nephritis, can not be questioned. Such remedies as spirit of nitrous ether and solution of ammonium acetate, and others, have their place; but for emergencies, pilocarpus stands at the head for efficiency and promptness. Depression, it is true, may accompany its action ; but used with caution and in cases of uremia, with a strong and tense pulse, it is capable of changing the condition of the patient from one of imminent danger to one of comparative or complete safety.

I have already referred to the good effects of cholagogues. It is hardly necessary to mention the value of hydrogogue cathartics and salines, both in acute and chronic cases, and to invoke their aid when dropsy occurs, or when diuretics and diaphoretics are contraindicated or fail in their action.

Nitroglycerine is oftentimes invaluable. In acute cases of nephritis, with high pulse tension and diminished flow of urine, its place can not be easily supplied; while in chronic interstitial cases its influence upon the general and upon the renal circulation is equally pronounced. The tolerance of this drug by many patients is noteworthy. When normal pulse tension fails and the heartchambers dilate, the resulting passive congestion is to be met with drugs which force the blood through the kidney. It is here that digitalis is preeminent. In my own experience it has no equal, and there are few patients to whom it can not in one form or another be safely and successfully administered. Its substitutes, however, may be necessary and are not to be underrated.

Although the measures used in chronic nephritis are more hygienic than medical, there are certain drugs, outside of those employed in complications, which serve a good purpose. Among these iron stands preeminent, especially when anemia has set in and the blood-vessels need to be nourished and strengthened. As to the particular preparation of this agent, I confess to a growing confidence in the old tincture of the chlorid of our fathers and grandfathers. Much depends upon the dose and its combination with correctives and synergists. Bichlorid of mercury, dilute hydrochloric acid, and liquor of the chlorid of arsenic are valuable auxiliaries, whose action, whether singly or in combination with iron, are oftentimes forcibly demonstrated in interstitial cases. In parenchymatous nephritis, with anemia, dropsy, and deficient urinary excretion, the time-honored Basham's mixture can hardly be dispensed with, as it is both a reliable hematic and diuretic. It is encouraging to observe pale urine becoming normally colored under the use of iron, this effect being one which I always desire and endeavor to obtain, and which was very noticeable in one of the young children to whom reference had been made. When the tincture fails, other preparations of iron can be used with advantage.

In anemic convulsions none of us doubts the efficiency of chloral hydrate and the bromides, but there may be those who would be disinclined to use morphia. For my own part, I do not hesitate to invoke its aid. It is the only drug which in certain cases will put the physician in command of the situation, and the patient is entitled to the relief or alleviation which it secures. I have never had reason to regret, on account of injurious effects, the use of morphia when circumstances seemed to demand its employment. That it may do harm is undeniable, but unpleasant effects can be prevented by not giving too large a dose. Veratrum viride is a remedy with which my experience is limited, although its virtues as a reducer of pulse tension and an internal bleeder are well established. In acute inflammatory conditions, with a strong pulse, the good results obtained from aconite must not be forgotten.

For the debility which sooner or later appears in all cases of sub-acute or chronic nephritis, recourse must be had to stimulation. The selection of the agent for this purpose requires discrimination. On account of the physiological relation of urea to ammonia, it is questionable whether the salts of ammonium may not, if too long continued, increase the production of urea—a result which is far from desirable when the secreting epithelium of the tubules is partly or wholly destroyed. Under these conditions a fatal termination is not far off; nitrogeneous food itself makes the patient worse; and any drug which causes tissue waste or increased formation of urea in the liver is, if possible, to be avoided. Alcohol, so often the first cause of nephritis, may be beneficial, especially in elderly people who are the subjects of gradual degeneration of the kidney. It is best to give it with food. In other cases, however, its regular use as a stimulant can not fail to react upon the system and intensify the symptoms which it may temporarily appear to relieve. Strychnin may be contraindicated in excitable states of the nervous centers, but in such conditions the pulse is usually tense or strong, and the drug would not be considered. Many are the times when it can be given with propriety and marked benefit. When the heart fails and the pulse grows soft, thin and frequent, it is a powerful ally of digitalis. Taken all in all it is the best remedy in our possession for weakness and exhaustion of the nervous system, while its combination with the hypophosphites will sometimes produce results unattainable by either drug alone.

In conclusion, it is needless to add that it has not been my purpose to treat the subject exhaustively, nor to discuss fully specific articles of food and specific drugs. On the contrary, my aim has been to call attention to the underlying principles which should be observed in treating nephritis with diet regulation and medicinal agents. The leading drugs have been mentioned in order to illustrate principles, and with the same idea in view, reference has been made to certain important articles of food. If what has been written will stimulate to a deeper study of the physiological action of drugs and of the metabolism of food products in the body and lead to a renouncement of that skepticism on the subject of medicinal therapeutics which is growing too common in our ranks, I shall be abundantly rewarded for the privilege of reading this paper.—*Courier of Medicine.*

### PERSONAL EXPERIENCES IN ANGINA PECTORIS.

BY M. B. SMITH, M.D.

As practicing physicians we see many cases of incurable or serious disease living in comparative happiness because of ignorance of their true condition. Often the saddest thing in a physician's experience is to see a brother practitioner stricken down with some disease which he realizes must end fatally. It is one of the unfortunate sides of a physician's knowledge.

Curiously enough, however, much of the medical knowledge of the day has come from the work of physicians on their own cases, or from the impetus they have received from the fact that they were sufferers from certain diseases. It is a well-known fact that diseases have lain undiscovered and unsuspected until diagnosed rightly by some suffering physician. An excellent illustration of this fact is seen in the case of so-called "hay fever" or "autumnal catarrh," which was first described by Dr. John Bostock in 1819, in the tenth and fourteenth volumes of the Medico-chirurgical Transactions, under the name of "autumnal catarrh." Bostock, attracted to this disease by his own sufferings, collected twenty-eight similar cases, ten in his own observation, and from these reports arose the conception of a new disease or condition. Again, in this same disease, Dr. Morrill Wyman, professor of theory and practice of medicine in Harvard University, described, in 1854, another form of catarrhal trouble occurring in the spring, which he termed "rose cold" or "June cold," which seemed to him different from the "hay cold" or "hay fever." Nowadays the profession is inclined to believe that these divisions are superfluous.

Cases are on record where physicians have diagnosed their own diseases, even in such insidious mental conditions as paresis. Chase, in his recent work on "General Paresis," cites a case where a physician decided correctly that he had paresis, only to lose later his conception of his own case in the deterioration occurring in this disease. Haussman, a German physician, a few years ago described his sensations and experiences occurring during an attack of diphtheria, and especially during the diphtheritic palsy which happened to follow in his case.

Coming to heart cases, we find many illustrations. One of the most famous is that of Dr. Hiram Corson, of Conshohocken, Pennsylvania, who practiced over sixty years in the hardest sort of country for exposed work, and who suffered all this time from attacks of what was diagnosed by Professor Horatio C. Wood as "cardiac nerve storms." Despite these attacks Dr. Corson lived to a very ripe old age, as the writer can testify. I spent a day with Dr. Corson after he had been in the practice sixty years, and his vigor, both mental and physical, was far above the average of men half his age. He drove down the steep hills of his region with the utmost recklessness; or rather he allowed his horse to run down, for he threw the reins on the floor of his buggy, and, laughing at my expression of alarm, said he spent his hours in his carriage reading, allowing his horse to depend on his own agility to save his neck and that of his master. It seemed to be a good plan, for Corson did it for sixty years, and finally died in bed.

I wish to describe my own experience in angina pectoris, as I believe it will be useful to many practitioners, for the text-books do not give details enough to cover these cases intelligently. John Hunter, a sufferer from this disease, was wont to say, "My life is in the hands of any rascal who chooses to annoy and tease me," and he subsequently died during a fit of anger.

Of course we must realize at the outset that angina pectoris is only a symptom, as dropsy is a symptom, and due to many varying causes. There are many morbid conditions of the heart and vessels which will produce angina. Generally these conditions are associated with sclerosis of the root of the aorta and changes in the coronary arteries. Osler believes that true angina is a rare disease, but such has not been my own experience.

Again, it is a disease of adult life, occurring generally in men. Huchard collected two hundred and thirty-seven cases, of which only forty-two were women; while in a series of Osler's cases of forty only one was a woman. Angina, according to the text-books, comes on frequently following exertion, such as walking up hill, or sudden muscular effort. In my experience it is far more apt to come on owing to a long-continued strain, which in itself may seem slight. I will cite an instance to illustrate my point. A well-known man, apparently in superb health, suffered from angina severely. He was under care of his physician for years, and lived a quiet life, but undoubtedly the angina was due to an exciting tragedy in which he had figured some years before, during which he was under prolonged mental strain. Staying one summer in the country, he had several slight attacks of angina, and consulted with a country practitioner about the advisability of driving to town, a distance of ten miles. The country doctor, looking at his magnificent physique, at once advised that it would be perfectly proper. He examined the patient's heart, and to him the story of angina seemed a myth. The man drove to town with a peculiarly hard-mouthed horse, and on reaching his door-step, tumbled over dead. Undoubtedly the steady strain for two hours threw the heart and its arteries into such a spasm that it quenched the man's life. If he had stayed quietly in the country until the attack was over, he might have lived indefinitely. The country physician, on hearing the result of his advice, was astounded. He said at once: "I have practiced for thirty years, and I will acknowledge I know nothing about angina; the man seemed in superb health; there was no heart lesion; his appearance was calm, his heart-beat steady and quiet, and it looked to me like a man who was simply nervous over nothing."

Again, violent sustained work coupled with mental anxiety is peculiarly liable to produce angina; I have found that many old football-players have the disease. The exertion and the anxiety work together with dire results. Latham makes a distinction in the paroxysm; the pain or *dolor pectoris*, and the indescribable feeling of anguish and sense of imminent dissolution—*augor animi.* The pain may be of any sort at all, sometimes radiating up the neck and down the arm, with numbness of the fingers. Generally it is a sensation as if steel fingers were clutching the heart, as is somewhat graphically pictured in advertisements in the medical journals. This paroxysm may be so short that it seems like a flash of electricity, or it may last a minute or two. It is in the latter cases that the sense of death is generally imminent. While there is great restlessness, there is also curiously a great longing for intense quiet of body and mind; the patient hates to see or hear bustle or confusion. A feeling of exhaustion or even collapse follows, lasting even several days. Head and Mackenzie have tried to study the radiation of this pain, and conclude that in diseases of the heart, and more particularly in aortic disease, the pain is referred along the first, second, third, and fourth dorsal areas, while in angina it is referred along the fifth, sixth, seventh, and even eighth and ninth dorsal areas.

There are many theories as to the cause of this pain. Some believe that there is a true neuralgia of the cardiac nerves. The sudden cramplike character of the pain and the suddenness of the attack are not especially like neuralgia elsewhere. Other authorities think it is a cramp of the heart muscle itself, or that it may be due to the extreme tension of the ventricular walls associated with sclerosis of the coronary arteries. The "Allen Burns" theory is that the condition is one of transient ischemia of the heart muscle in consequence of disease or spasm of the coronary arteries.

I will now take up my own experiences in the disease. In the first place I have no drug or no treatment that seems to be of value in the disease. Years ago I tried the use of nitrite of amyl as originally advised by Lauder Brunton. In my case it made me feel ten thousand times worse; the pain may have been relieved, but there was a sense of impending death which the drug made worse, and a fulness of the head that was simply terrifying. I have found the attacks went off as well without the drops as with them, so I speedily rejected this form of treatment. There is a relaxation of the heart and its contractions brought about by nitrite of amyl that seems to me almost as dangerous as the opposite condition. I then tried other drugs and other treatments, but practically they were *nil* in their results.

Hence I am forced to this conclusion : *the way to treat angina pectoris is to make the patient live in such a manner that the possibility of the attacks is decreased.* I believe no drug or treatment does much for the attack; therefore when you have a sufferer from angina under your care, it does not pay to temporize. Get that patient into a method of life that will help ward off these attacks. What do the statistics of Huchard and Osler show, with practically no women in their lists? It means that angina is due to anxiety and strain. It is an expression of "strenuous life," so widely advertised and advocated by our writers of the day.

As a rule you will find these sufferers are busy business men with heavy anxieties; those strains must be removed. The utmost objection will be made at once by these men: "We can't stop;" "We have families to support;" "We'll stop next year," etc. It will not do; your patient must lead a quiet, comfortable life, getting plenty of sleep, good food, and exercise. It is here that golf I believe is of use; many men have found it has cleared their brains, stimulated their hearts, and removed anxieties. This seems a simple thing to do, to remove a patient from his sources of trouble; but it will tax the courage, the ingenuity, and the honesty of any practitioner. I say honesty, for it is so much easier to shut our eyes and let things go on as they are.

There is another feature which I have seen mentioned in no text-book or article on angina. It is that angina changes the character of its victim: for example, instinctively such a man will avoid the things which produce these symptoms until it will change a combative man into the veriest coward. I have found that anger or indignation is my special *bête noire*, and I have gone far out of my way to avoid unpleasant people, unpleasant sights, or unpleasant duties. I have let things pass that I should have taken up, because I knew it would produce angina. Even the envelope of a letter which may contain unpleasant news I hate to see. I find that I must avoid strain and strenuosity, and it has been a great detriment to my progress, for it may convince my neighbors that I am sleepy or unprogressive. But in this way I have cut down my attacks of angina in fifteen years so markedly that I never now have more than rumblings of the disease, having had no real attack for several years. Fifteen years ago I had them frequently, some of them being so bad that I could not lie down, but was propped up in bed with anxious-eyed watchers around. Now I would pass muster in any physical examination, and am considered by every one to be in perfect health.

I avoid the people who irritate and annoy me; I cultivate those who stimulate me pleasantly; it is absolutely vital. I have been compelled to gave up surgical work, for several times angina came on during operations; and I have found obstetrical work very bad for me. This may be different from other cases; I am citing simply my own.

Another phase angina has produced in me is that of temper. As I have said, I will go miles to get out of the way of unpleasant people, but in the past I have found it occasionally impossible. Then realizing that I was in for an attack of angina, I have felt so outraged that any one would jeopardize my life, even unwittingly, my anger has been excessive. I have surprised greatly every one who has had any unpleasantness with me by my vigor when aroused. I do not seem to be able to control this; my reasoning powers are opposed to it, but it seems as if the paroxysms which come on at this time produce a state of almost delirious excitement. Afterwards I react terribly, and am unfit for work for a week at least. I had no experiences before angina developed, and in the past few years have been able to control such feelings better, largely by avoidance of sources of annoyance.

I believe that a case of angina pectoris caught early in its progress can be indefinitely held up by careful co-operation of patient and physician by such treatment as I have outlined. After fifteen years in the condition, I am in a thousand times better shape by the simple use of a definite plan.

In closing I would only say one word as to rest. I am a great believer in the theory that our bodies are after all only storage batteries containing a certain amount of force. This can be used up slowly or quickly. Dr. Wm. Pepper died at fifty-six, after having done a life's work which he might have prolonged to seventy-five or eighty. And by rest I mean rest in the recumbent position. Lying down, the heart's work is simpler and easier, and this in seventy years may be a great factor. Rest as much in the recumbent position as possible during the day.

Surely my treatment of my own case has been a success. I would rather be alive and live along quietly and unassumingly than be dead with a big notice in the *Journal of the American Medical Association.*—*Medical Age.*

---

RECTAL SURGERY IN THE GENERAL PRACTICE OF MEDICINE.*

With us of Henderson city and county it is a duty to do each for himself more or less surgery. We are fortunate in having among us men of special skill, and men who have, in certain lines of general surgery, developed ability to do some things better than others. The same is true in certain lines of general practice. Perhaps no one among us has so thoroughly mastered all branches as to stand, like Saul, head and shoulders above the rest.

The object of our effort is to give the best service to suffering humanity. A thorough understanding of the strong points in the individual make-up of our ranks, and a liberal use of these superior qualities by frequent consultations and honest dealing with each other and our patrons, can alone secure this chief object.

True as this may be, we go back to our first statement—we must each for himself do more or less surgery. Each must incise abscesses, dress wounds, treat fractures, reduce dislocations, etc. We do these things because we can do them, and do them well, and because we should do them. The plea of this paper is that rectal surgery should be placed in this class of surgery and be done by the general practitioner with the same degree of skill and confidence as he does any of the commoner operations of his craft.

I quote a familiar author: "It is a well-known fact that diseases of the rectum have not received that careful attention by the medical profession which its importance demands. From time immemorial these ailments have been in the hands of the charlatan." Much as has been done and written on this subject, this language is as true to-day as when it was written by our own Dr. Mathews, a decade ago.

*Read by D. O. Hancock, M.D., Member of County, State and American Medical Associations, Henderson, Ky., before the Henderson County (Ky.) Medical Society, October 12, 1903.

These truths bear causal relation to the existence in every community of a number of unfortunates who have easily curable affections of the rectum. There is hardly a man or a woman but knows of cases, and can give you names and residences of persons so afflicted. The charlatan has shown himself so unskilful and so unscrupulous, both in treatment and in fees, that our friends dare not trust him, choosing "rather to bear the ills they have, than to fly to others they know not of." We all remember a familiar figure going about our streets not long since, who, after confinement in bed for several weeks, carried an air-cushion about with him for quite a while. All was the result of improper treatment at the hands of a man who "cured without the knife."

The family physician who can not, or will not, properly treat these cases, but who prescribes (frequently without making an examination) ointments, salves, astringent lotions, etc., deters these patients. Many have, and rightly so, too, the utmost confidence in their "family doctor." What he does is all that can or need be done. Therefore, they go on suffering.

Again, the rectal specialist, trained in New York and in London, must have for his services fifty or one hundred dollars if it be a poor man, and upward *ad infinitum* if the patient be rich. The patient must also pay traveling expenses and sanitarium bills, making a cure at the hands of the specialist too expensive for most people. Besides, the family doctor has a confidence, which long years has established. This the specialist can not have. At the earnest solicitation of the home doctor and after torture unbearable, the patient may go to the specialist, but then with fear and trembling.

The practitioner should do this work because: First, he has the confidence of his patient and can persuade him to submit to operation. Second, he can do this work at a place convenient to the patient and at a cost which the man can pay. Third, he *can* do this work, and do it well.

With ability, economy and confidence at our command, the long sufferers from piles, fistula, fissure, stricture and constipation should disappear from among us, and no longer advertise our incapacity and indolence. No refinement of specialism will ever relieve us of our duty to these patients.

I will stand corrected if the sense of this presence is that more than 50 per cent. of these cases of surgical rectal diseases occurring in our territory are treated successfully by us. I verily believe that a large per cent. of the physicians of Henderson county have never done a surgical operation upon the rectum. By this I mean not that they have not split a blue external tit, evacuating a clot, or clipped off a tag of inflamed redundant skin, or, under cocaine, tied off an external pile, which, being inflamed, seemed to the patient as large as a walnut, or incised an abscess, or cauterized a fissure; but I mean that under general anesthesia, to divulse the sphincter, and by one of the recognized and only ways of curing these cases, do a surgical operation upon the rectum. Again I stand corrected, if I have stated this too strongly. Of this estimated 50 per cent. of cases that have not submitted to surgery at our hands, some have gone to the specialist. For them we have not a word of complaint. Some have gone to the charlatan and shark. They have our sympathy. The remainder go about among us, in agony of body and mind, incapacitated for business or social life. Many doctors of our county do this work successfully. I have estimated that 50* per cent. of these cases are cured by them. But I repeat: These affections should be placed in the same category with fractures, dislocations, contused, incised and gun-shot wounds, and treated by every practitioner with the same confidence, skill and success. I am not unmindful of the advantage of having a "sure-enough" surgeon in all of these cases—one who gives his whole time and thought to surgery. But such person is not available or practicable in the country, or in towns, or in small cities. In large cities they are often out of the way or engaged, and the family doctor must do surgery.

Reflexes have, in recent years, occupied a large place in medical literature. Those of the rectum are many. Often we are first consulted for some reflex, and by our familiarity with the subject we are able to locate the trouble, and by surgery relieve it. We

*Too great. Dr. Dixon thinks 10 per cent. large enough.—Discussion.

pass also the etiology of these diseases. It must, however, be understood, if we would render our patients the best service.

I shall not affect an original method of dealing with any of these conditions. I would not advise that the general practitioner undertake such surgical finesse as submucous ligation of hemorrhoids, or denuding a pile of its mucous membrane, dissecting out the hemorrhoid, and after controlling the bleeding, by tortion if necessary, replace the mucous membrane, and get union by first intention. Nor should he attempt to dissect out a fistula, and, securing apposition with sutures, get the wound healed over in a week. There are methods of operating which are classical. They are recognized as safe and sure of cure. Let us stay hard within these lines. One of these mastered and practiced will give satisfactory results.

HEMORRHOIDS.

In treating this part of our subject, we assume that every physician can make a diagnosis. It matters little if external piles or internal piles, itching piles or blind piles, raspberry piles or *gooseberry* piles. When the sphincter is divulsed, and the pile area everted, you have all of them external piles, and you treat them as big piles and little piles; the clinical importance of which is that the large piles must be transfixed, if treated with ligature.

Owing to the absence of valves in the hemorrhoidal veins, and to the erect position of the body, and to the peculiar office of the rectum, these veins are very liable to become dilated and varicose. They may contain a blood clot, yet neither, nor all of these conditions constitute piles. The treatment, as laid down for hemorrhoids, whether palliative or operative, could not be properly applied at this time to this condition. Here, doubtless, is where the treatment of piles by the dorsal decubitus, with hips elevated, as advocated occasionally, comes in. Here, also, is possibly where cures "without the knife" are most frequently accomplished. This preceding condition tends to and may culminate in piles. There must, however, be a further pathological change to constitute the hemorrhoidal affection. The congestion induced and the friction necessitated cause an inflammatory exudate to take place in the tissues, and a veritable tumor is the result. This tumor is well defined. It can be seen and handled, is firm to the touch, and grows by plastic infiltration. Remove it and you cure the condition. This, possibly, may be done by absorption, but such is very rare, if at all. We must cut them away, burn them away, or slough them away.

*The injection method* of treatment contemplates either absorption or sloughing. The one by limiting the blood supply and stimulating absorption; the other by cutting off the blood supply by caustic injections, causing sloughing of the parts. The first is inconsistent, the second irrational. Both are dangerous. Frequently gratifying results are obtained by each.

*The cautery method* is thought by its adherents to be the ideal operation. The gut is clear at once. There are fewer reflexes, less after-pain, and quicker recovery. There is, however, greater danger of secondary hemorrhage, and more liability to recurrence, because the dilated, varicose condition of the veins that frequently exists as pre-hemorrhoidal tissue can not be so well removed without incurring risk of a strictured condition of the bowel by excess of cicatricial tissue. It is a standard, classical method. Those who practice it do safe and satisfactory surgery.

*The ligature method* combines removal by cutting and sloughing. I operate by this method, and prefer it, because during my time in medical college and my year as interne I saw no other performed. Results from these were uniformly satisfactory. Because, also, I am familiar with its technique, and have never been disappointed by its use. I believe it the safest, as to life, the surest of permanent cure, and freest from accidents.

Our text-books describe these operations better than can be done in this paper.

*Abscesses* about the rectum demand more attention than elsewhere. They should be freely incised and constantly looked after. If large, it may be necessary to give a general anesthetic before incising them, which should in every case be done early. If possible, prevent fistula.

### FISTULA.

This affection, I believe, is of less frequent occurrence than hemorrhoids, but of more serious import. Fistula kills. No temporizing should be indulged. Delay only makes more difficult a cure. A simple sinus, with no cicatricial tissue or tube (if such ever occurs), may be cured by cleansing and the use of the fistulatome, or by injecting irritants and antiseptics, or by the elastic ligature—all of which are unscientific and unsurgical and of doubtful efficacy. It is impossible to know that there is but one track. The incision will enable us to know by searching with the probe if there are multiple tracks, and if there are not, it is still the most surgical way of treatment. I therefore believe that for fistula there is but one treatment. Follow up the track, or tracks, to their last ramification, with a grooved director, and cut straight out. Dissect out the bottom of the tract, and treat an open wound. The general practitioner should not only not attempt apposition and union, but he should vigilantly prevent such occurring. There is great disposition of the parts to bridge over and close out the job. I have often pulled the adhered surfaces apart, and sponged firmly in the bottom of a rapidly healing wound of this kind. Insist that the wound heal from the bottom, by healthy granulations. Then, if you have been successful in finding and dividing all the sinuses, you will have a permanent cure. If a single sinus has been overlooked and left, most certainly there will be a recurrence of the trouble. Improper after-treatment may also cause a recurrence. It is sometimes impossible to go to the end of every sinus. This should be recognized at the time, and explained to the immediate friends. Thereby we shall avoid criticism when recurrence shows itself. It is wise, also, to explain the extreme difficulty there is in finding every track and the probability of overlooking one or more of them; so that if the cure is not effected, you will retain the respect of your patron. I have known a good man to make this mistake, and be the subject of comment for years thereafter. I may not suggest more for this operation than is found in descriptions of it in our text-books. I shall not classify, as we find it in our books. Clinically, it matters not if the fistula is a blind one, or has two eyes; or one outside or one inside, or a horse-shoe or a mule-shoe. If an abnormal hole is found, follow it to the bitter end, having respect, of course, to certain muscles, blood-vessels and organs. If this can not be don e, palliative operation may be performed, and the explanation suggested made to the friends.

*Fissure,* sometimes called irritable ulcer, is perhaps the most painful of all these affections. This may be treated medically, but far better surgically. A number of operations have been proposed. A satisfactory manner of dealing with this trouble is to anesthetize the patient, divulse the sphincter and scarify the ulcer. If there is much induration, it is well to dissect out the fissure and ulcer, and either suture the mucous membrane, or, better, leave it to care for itself.

*Ulcers,* large and small, are frequently found in the rectum proper. Divulse the sphincter, and curette. Then treat with injections—antiseptic, astringent, emolient.

*Stricture* is the most obstinate of the curable diseases of the rectum. Give chloroform, divide dorsally, divulse, put in a tube wrapped with gauze, which remove after three days, and treat with antiseptic injections and oils. Reformation must be prevented. The cause, if ascertained, must be given consideration.

The surgical treatment of *constipation,* by removing valves of the rectum, is recommended by so eminent an authority as Dr. Gant, of New York. It is well to leave these valves, their existence, their function and their treatment, yet a while with the college men.

When every doctor has learned to treat, surgically, these conditions, and to diagnose and treat the medical diseases of the rectum and sigmoid flexure successfully, there will be less suffering in the world, more money in the doctor's purse, and more grateful hearts to bless him and his labors. I have purposely not essayed cancer of the rectum and sigmoid flexure, or tuberculosis of these parts. I shall not enter the discussion of the extirpation of the rectum, or of the propriety of colotomy. These graver conditions and more serious operations I regard as outside of the duty and reasonable skill of the general practitioners.

The treatment of these affections at the doctor's office is advocated by good men. The milder forms, and even the large pile areas, are being cleared in segments, by using local anesthesia. Some of us, however, prefer to limit the use of cocaine in our office work. The power of suggestion works better on other parts of the body than on the rectum. Patients do not court pain at our hands. Office treatment may be successfully done by the specialist and men of great reputation, but for the general practitioner I think it wisest to give chloroform or ether before any attempt is made at surgery, and then do a complete operation.

The subject of *anesthesia* is of such importance as to demand especial and separate consideration. A most excellent paper on this subject was the oration of Dr. Lewis before the Kentucky State Medical Society at its 1903 meeting. Remembering the reflexes, it is highly important that the patient be fully under the anesthetic before divulsing the sphincter. Should respiration become embarrassed, a positive stimulus will be given by divulsion.

*The preparation of the patient* for operation should cover a period of three days, during which time the diet should be restricted. On the first day, at night, give a full dose of calomel; on the second day give saline in the morning and full doses of quinine in the afternoon; on the third day give quinine in the forenoon; on the fourth day give enema in the morning, early, and also one hour before operating, say at 10 o'clock a.m.; have patient remain in bed on the morning of the operation.

The after-treatment should be rest in bed and morphia to control pain and prevent the bowels moving for three days, when an enema is given and such subsequent treatment as the case demands.

We have not gone far into any division of the subject. If these suggestions shall assist in the selection and use of a safe way of treating these diseases I shall be amply repaid. The burden of this writing is an effort to get at some means of reaching neglected sufferers. I have endeavored to place the responsibility upon the general practitioner and urge him to do his work.—*Louisville Journal of Medicine and Surgery.*

---

***NOTICE.— This Journal and the International Journal of Surgery one year for $1.00 cash, to new subscribers. No out-of-town checks accepted unless 10c. is added for cost of cashing. Strictly to new subscribers.***

### REPORT OF CASES TEATED BY TERRALINE.

BY E. C. ROEMELE, PH.D., M.D.,

Lecturer on Materia Medica and Therapeutics in Hospital College of Medicine, Louisville, Visiting Physician to Paint Mission Infirmary, etc.

CASE 1. Carrie S., aged 19, occupation stenographer; diagnosis, lobar pneumonia, presenting the following symptoms: Began very suddenly with a chill; a dry, hacking cough set in and the characteristic brick-dust sputum was present, as was also the pain in the side. All the regular symptoms of pneumonia were present.

*Treatment:* She was at once given the following:

℞ Hydg. Chlor. Mite.......................................gr. ii.
Sodii Bicarb.......................................gr. vi.
Pd. Ipecac.......................................gr. i.
Mft. Pulo. No. 6. Sig. One every hour.

Also, the following cough mixture:

℞ Ammon. Carb.......................................ʒ i.

Syr Tolu.......................................℥ ii.

M. Sig. Teaspoonful every two hours.

Although she had taken the above for several days, the cough did not become loose and great pain was experienced upon coughing. This form of treatment was then discontinued and the following given:

℞ Terraline.......................................B i.
Sig. Teaspoonful three times a day.

Under this medication she immediately began to improve, the cough ceased, the lung underwent complete resolution and the patient made, not only an uneventful, but most remarkable recovery.

CASE 2. Geo. F., aged 22, occupation brakeman. This young man is the only one left of seven children, the rest all having died of phthisis pulmonalis. The writer was not the attending physician in any of the other cases, but, some eight months ago, this young man called upon me, complaining of cough, especially during the night. After getting his family history there was absolutely no doubt as to the diagnosis (phthisis). He was at once placed upon various preparations of cod liver oil and creosote. After taking these for several weeks his stomach become so deranged that he suffered anorexia and neither food nor medicine could be retained. Of course this treatment was discontinued. He was then placed upon terraline in tablespoonful doses. His stomach at once improved, his appetite becoming better, his cough began to improve, so that after two months' treatment it had entirely ceased; however, treatment was continued until two months ago, when I presented him before the medical society and no one present could detect any signs whatever of consumption. I am thoroughly convinced that in this case one of phthisis pulmonalis was cured by terraline. There has been no return of symptoms whatever.

CASE 3. James F., aged 30, occupation carpenter; diagnosis, pneumonia. A very heavy drinker. Was brought to the infirmary on about the fifth day of his illness in a delirious condition. He had been sent to us (because he was not expected to recover) by the physician who attended him at his home, who, of course, gave up the case. The writer did not know what treatment had been given him, but at once ordered a hypodermic of morphia 1/4 grain, and a tablespoonful dose of terraline four times daily. Hot applications were applied to his chest. He passed a quiet night. The following morning the temperature was 107½ F., pulse 140. He was given the ice cap. Strych. sulph. gr., 1/30 three times a day in conjunction with the terraline. Within ten days the man left the infirmary sound and well. I attributed his recovery to the terraline simply because I afterwards learned that he had been taking all the other treatments by the physician who sent him to our infirmary.

CASE 4. Eugene K., aged 7; bronchitis. Had been under the care of a homeopath for three weeks but without any good results. The writer was then called in consultation and found the case to be a typical one. I ordered a dose of castor oil and the following:

℞ Terraline.......................................... ℥ viii.
Sig. Teaspoonful every three hours.

Within one week the child was entirely well.

CASE 5. W. S., aged 9; bronchitis, presenting the following symptoms: Pain in back, headache, vomiting, very irritating cough. Temperature, 102. He was given the following:

℞ Hydg. Chlor. Mite.....................................gr. ii.
Sod. Bicarb...........................................gr. viii.
M. Ft. Pulv. 8........................................
Sig. One every half hour.

℞ Terraline...........................................℥ss.
Sig. Teaspoonful every three hours.

After eight days he was entirely well and allowed to attend school.

CASE 6. D. A., aged 30; diagnosis, consumption. Had been under the care of several physicians, had also consulted advertising physicians, had gone to Colorado in hope of regaining his health and was told that he would not be able to live in Kentucky two months. He decided that if he were to die he would die at his home (Kentucky). He came to the infirmary weighing 103 pounds. He was placed upon absolutely nothing else except terraline, tablespoonful doses three times daily. When he came to the infirmary he could retain absolutely no food. However, after taking several doses of terraline he could eat as well as ever and began to improve in every respect. After he had been under my care two months (his alloted time to die) he said: "Well, Doc., I am far from a dead man." He gained in weight and after three more months' treatment weighed 130 pounds, cough disappeared and he said that he was well. He was discharged two months ago and since then has been working daily, continuing to remain in perfect health.

CASE 7. Baby F., aged two years; diagnosis, bronchitis. Was given castor oil and half teaspoonful doses of terraline, made a complete recovery within seven days.

CASE 8. Jas. B., aged fourteen; chronic bronchitis. Came to the clinic stating that he had been under the care of doctors for three weeks and had taken various kinds of medicines but got no better. He feared he had consumption. After I had examined him thoroughly I found he had chronic bronchitis. Gave him two grains of calomel and terraline in teaspoonful doses every four hours. He made a complete recovery within ten days.

CASE 9. Ben T., aged twenty-eight; asthma, presenting all the symptoms. Was unable to lie down to sleep; came to the clinic stating that he had been under the care of the family doctor but got no relief. He was given terraline, tablespoonful every four hours. He came back two days after stating that that was the first night in one year that he slept in bed. After four weeks he said he was well and never again returned to the clinic.

CASE 10. John B., aged twenty-four, carpenter; diagnosis, lagrippe. Had been sick two weeks under the care of another physician. His cough was most irritating. The writer was called in consultation and found that he was getting the following treatment only:

℞ Quiniæ Salicy.

Phenal Salicy ........................................a a ʒss.

Caffeine Cit ..........................................grs. x.

M. Ft. Caps. 10.......................................

Sig. One every three hours.

This treatment was continued, but terraline, in tablespoonful doses three times daily, was prescribed. The patient at once began to improve and was able to attend to his business within twelve days. I might also mention that a case of indigestion which the patient had before his illness was also cured by terraline.

CASE 11. O. G., aged seventeen; severe cold. Was given terraline in teaspoonful doses every three hours and made a complete recovery in four days, the cough disappearing on the second day. He had been taking cough mixtures before without any beneficial results.

CASE 12. Wm. F., aged nineteen; diagnosis, phthisis pulmonalis. Under care of another physician. The cough became so severe that the family requested that I be called in consultation. I found that the patient was in the very last stage, having been in bed for three years. He was given terraline in tablespoonful doses three times a day, which relieved his cough, enabling him to sleep at nights and increased his appetite wonderfully. After two months treatment he was able to sit up in his room and two and one-half months later he was able to work about some, when he was sent to Mexico.

CASE 13. James E., aged 26; diagnosis, pneumonia, presenting all the regular symptoms. Was given the following:

℞ Spts. Aetheris Nitrosæ....................................ʒi

Potassia Acetatis...........................................ʒii.

Aq. Camph....................................................℥iii.

M. Sig. Teaspoonful every three hours.

℞ Terraline.............................................. Bi.
Sig. Tablespoonful every four hours.

Made a complete recovery after two weeks' treatment.

CASE 14. Mary R., aged thirty; diagnosis, asthma of three years' duration. Had been taking patent and other kinds of medicines. When I called I found her just recovering from one of the paroxysms, which she said she had about every two hours. I at once gave her terraline in tablespoonful doses to be taken just before she felt one coming on her. The paroxysms decreased in number until, at the present time, she does not average one in twenty-four hours.

I could cite numerous other cases in which I have had equally good results with terraline, but space will not permit.

---

### ADRENALIN AND ITS USES IN GENERAL SURGERY.

Under the above title an article appears in the October issue of the *Indian Medical Gazette*, from the pen of Harry Gidney, F. R. C. S. (Edin.), D. P. H. (Camb.), etc. The author finds that "the clinical usefulness of adrenalin is very great and extensive, and owing to its power of rapidly and effectively producing vasomotor construction, it is adapted to the treatment of all inflammatory conditions. The drug is also of extreme value in arresting hemorrhage during all surgical operations. It is indicated whenever and wherever any local hyperemia exists, more especially in inflammations of mucous surfaces, such as those of the eye, throat, larynx, pharynx, urethra, bladder, nose, rectum, vagina, uterus, stomach, etc. It is used not only to stay hemorrhage when it exists, but also as a preventive or controlling remedy, given either internally or externally prior to an operation, so as to lessen the amount of bleeding during the performance of that operation. It is a non-irritant to mucous membrane, unless when used too frequently and to excess.

"On reading the literature on the subject," says the writer, "I find that adrenalin is admitted to be the most powerful and rapid cardiac stimulant and tonic we have, being chiefly used in cardiac affections, hematemesis, hemoptysis, hemophilia, hematuria, menorrhagia, post-partum hemorrhage, purpura, scurvy, etc. It is said to be the most rapid restorative in chloroforms and other forms of anesthetic syncope, and in such cases it is advisable to administer it intravenously."

The author reports the results of several operations, major and minor, in which adrenalin was employed. The first case was one of fracture of the vertex of the skull. As one of the larger branches of the middle meningeal artery had been torn, there was profuse dural hemorrhage and capillary oozing, which were controlled by the use of the 1-1000 solution. In the second case, one of hemorrhoids, profuse bleeding was checked by the rectal insertion of a plug of cotton wool soaked with adrenalin chloride solution.

The third case was one of skin-grafting, in which the author tried pressure to stop the capillary bleeding. As the procedure was somewhat tedious he applied adrenalin chloride solution with almost immediate cessation of all oozing, and what is usually a lengthy and sanguinary operation was converted into a short and comparatively bloodless one.

The fourth case, one of hemorrhage after the extraction of teeth, and the fifth, which appears to embrace the author's experience in a number of cases of epistaxis, afforded additional opportunity to test the hemostatic effect of adrenalin.

In case six a post-partum hemorrhage was checked by swabbing the uterine cavity with adrenalin solution, while the same happy result was obtained in a case of secondary hemorrhage following an operation for the relief of a mammary abscess.

The author has found that the instillation of a 1-5000 to 1-2000 solution of this drug reduces the inflammation and considerably cuts short the process of conjunctivitis. He usually applies it (diluted) over the inflamed parts by means of a soft camel's-hair brush. He always uses the preparation containing chloretone, which has a decided local anesthetic action, relieving much of the photophobia and pain. He is fully convinced of the power of adrenalin to arrest or lessen the bleeding that arises from the cut ends of the iris after iridectomy. He speaks highly of its efficiency in chemosis, cataract operations, evisceration of the eyeball, operations for ectropion, symblepharon and trachomatous pannus.

The author concludes that in all cases of minor surgery in which it is desired to arrest bleeding from any cut or exposed surface we have in adrenalin a most useful, powerful and rapid drug—one that is non-poisonous, non-irritant and non-accumulative, especially in operations upon the conjunctiva and eyelids.

---

### MEDICINAL TREATMENT OF GALL-STONES.

Richardson (*Therapeutic Gazette,* November) says that gall-stones appear to be the result of an infection, producing an inflammation of the walls of the gall-bladder and a reduction in the total solids of the bile, especially of the bile acids, with an increase of cholesterine. Similar results have been produced by injecting mercuric chloride into the gall-bladder of starving dogs, showing that bacterial infection is not necessary to produce a condition of bile favorable to the formation of gall-stones.

Analyses of bile from fistulæ seem to prove conclusively that cholesterine gall-stones are the result of a deficiency of glycocollic acid. "It naturally suggests itself that the prophylaxis of gall-stones is the administration of glycocholate of soda by the mouth, since it will then be absorbed from the intestine, entering the gall-bladder from the liver, and hold the cholesterine in solution." The possibility of dissolving gall-stones already formed has also been considered. Harley and Barratt inserted large gall-stones into the gall-bladders of healthy dogs and found that in periods of from six months to one year they had entirely disappeared. In other cases in which they produced a cholecystitis the gall-stones remained unaltered.

From these experiments it is evident that by the administration of glycocholate of soda it must be possible to dissolve gall-stones in the bladder, and even when cholecystitis is present glycocholate of soda is indicated not only as a prophylactic but as a solvent for stones already present, and that in those cases only in which there is occlusion of the gall-duct is surgical interferenee permissible.

